# Fungal Endophytes as a Metabolic Fine-Tuning Regulator for Wine Grape

**DOI:** 10.1371/journal.pone.0163186

**Published:** 2016-09-22

**Authors:** Ming-Zhi Yang, Mian-Di Ma, Ming-Quan Yuan, Zhi-Yu Huang, Wei-Xi Yang, Han-Bo Zhang, Li-Hua Huang, An-Yun Ren, Hui Shan

**Affiliations:** 1 School of Life Science, Yunnan University, Kunming, China; 2 School of Chemistry Science and Technology, Yunnan University, Kunming, China; 3 College of Food and Nutritional Engineering, China Agricultural University, Beijing, China; Universita degli Studi di Siena, ITALY

## Abstract

Endophytes proved to exert multiple effects on host plants, including growth promotion, stress resistance. However, whether endophytes have a role in metabolites shaping of grape has not been fully understood. Eight endophytic fungal strains which originally isolated from grapevines were re-inoculated to field-grown grapevines in this study, and their effects on both leaves and berries of grapevines at maturity stage were assessed, with special focused on secondary metabolites and antioxidant activities. High-density inoculation of all these endophytic fungal strains modified the physio-chemical status of grapevine to different degrees. Fungal inoculations promoted the content of reducing sugar (RS), total flavonoids (TF), total phenols (TPh), trans-resveratrol (Res) and activities of phenylalanine ammonia-lyase (PAL), in both leaves and berries of grapevine. Inoculation of endophytic fungal strains, CXB-11 (*Nigrospora sp*.) and CXC-13 (*Fusarium sp*.) conferred greater promotion effects in grape metabolic re-shaping, compared to other used fungal strains. Additionally, inoculation of different strains of fungal endophytes led to establish different metabolites patterns of wine grape. The work implies the possibility of using endophytic fungi as fine-tuning regulator to shape the quality and character of wine grape.

## Introduction

Endophytes, including fungi and bacteria, are symbiotic organisms that live within plant tissues or organs but cause no obvious symptoms of infection [1]. Endophytic fungi widely distribute in all natural growing plants [2]. During co-evolution, endophytic fungi and plants have formed a mutualistic and symbiotic relationship. Endophytes benefit their host plants in multiple ways, such as growth promotion [3], nutrients absorption [4], or providing available nutrients for plants [5], as well as increased adaptability to pathogens, pests and abiotic stresses [6–8]. In addition, some endophytic fungi produce compounds similar to those found in their host plant, and these fungi are potential sources for active compounds that may have medical, agricultural and industrial applications [9–13]. However, few studies concerned the biochemical response of host plant to endophytes. It had argued that the secondary metabolites presented in plants should be re-evaluated by taking into account the existence of endophytes [14]. For one example, the induction of a Chinese traditional medicine "dragon's blood" (loureirins) from *Dracaena cochinchinensis* will be greatly promoted by the coexistence of certain species of fungal endophytes [15]. The possibility that plant secondary metabolites could be shaped by endophytes have gained various supports. Generally, endophytes alter host plants’ metabolites may by ways of: i) directly synthesize and secret metabolites in plant [11,16–18]; ii) purposely secret signal molecules, and consequently cause complicated cascade bio-chemical reactions in plant [19–23]; and iii) participate in the process of host plant metabolism by producing and secreting enzymes to plant and exert functions in plant metabolic pathways [24–26]. However, the extents of endophyte contribute to its host plant in metabolites and the underlying mechanisms require further systematic studies. Obviously, clues that endophytes possible influence the metabolites of host plants provide possibilities to finely tune the composition of secondary metabolites in a target plant using tools of endophytes. This is of special interest for those plants that are used to produce organoleptic sensitive products, such as wine, coffee and others.

Grapevine is one of the most economically important fruits in the world, and a large proportion is used for wine making. The quality and character of grape are fundamentally determined by its biochemical compositions, which affected by both genetic and environmental factors [27–30]. In addition to genotypes, biotic and abiotic factors also affect the metabolite contents of grape [31]. Research on how abiotic factors influence grape quality have been broadly covered, and some of these achievements have been successfully applied in viticulture [32–37]. Regarding biotic factors, attention has been paid also to ambient microorganisms on grape quality such as pathogens, rhizosphere microbes [38–41]. The well-known "noble rot" wine is made from plant pathogen B*otrytis cinerea* infected berries, and special components in *Botrytis cinerea* infected grape berries had been detected [42]. Although endophytes are abundantly distributed within grape leaves [43], and have special intimate relationship with vines [44], their metabolic effects on grape are vaguely explained. In this study, eight strains of fungal endophytes isolated from vine leaves were high-density inoculated to leaves of wine grape, 4 weeks after bud-burst Their impacts on grapevine physio-chemistries were assessed both in berries and full developed leaves at berry maturity stage, as well as discussed the possible application of endophytic fungi as metabolic regulators in viticulture.

## Materials and Methods

### Plant and fungal materials

A regionally planted wine grape cultivar Rose honey (*V*. *Vinifera* L.× *V*. *labrusca* L.) with high adaptive abilities in Yunnan province, China, was used in this experiment. Vineyards location belongs to subtropical climate, with altitude ~1400 m, annual precipitation 987.5 mm, yearly average temperature 17.3°C and average illumination of 2079.2 hours. The vines are five years old, spur pruned, with density of 1.2 m between rows and 0.9 m between plants, and following the local standards of fungicide (products of fungicide: metalaxyl, mancozeb, difenoconazole and Bordeaux mixture) and pesticide managements.

Eight strains of fungal endophytes isolated previously from grapevines [45,46] were selected for treating vines. Features of all these fungal strains and the status within the treated vines were summarized in Table 1 [47].

**Table 1:** Endophytic fungal strains used in the experiment.

Strain ID	Genus	Original host grapevines	Features in host grapevines [45, 46]	Status in re-inoculated vines [47]
CXB-2	*Xylaria* sp	Cabernet sauvignon	Dominant; Wide	Infected and become dominant species
CXB-11	*Nigrospora* sp	Cabernet sauvignon	Dominant; Wide	Infected and become dominant species
MXN-8	*Chaetomium* sp	Rose honey	Dominant; Wide	Not detected but changed the fungal endophytes community of grapevine
HCXL-16	*Alternaria* sp1	Cabernet sauvignon	Dominant; Narrow	Infected and become dominant species
CXC-13	*Fusarium* sp	Cabernet sauvignon	Rare; Narrow	Infected but existence with minority
Y73-11	*Colletotrichum* sp	Yan73	Dominant; Narrow	Infected and become dominant species
HMC-7	*Alternaria* sp2	Rose honey	Dominant; Narrow	Infected and become dominant species
CXC-9	*Gibberella* sp	Cabernet sauvignon	Rare; Narrow	Not detected but slightly changed the fungal endophytes community of grapevine

Features in host grapevine, “Dominant” or “Rare”: means the fungal strains were dominant or rare existent species in their original host grapevines; “Wide” or “Narrow”: means the fungal strains were distributed in most or only certain cultivars of grapevine.

### Preparation of fungal material

Fungi were plate cultured on PDA medium for 5–7 days (depending on the growth speed of the fungus). Five medium-fungal mycelium discs were cut with an 8 mm diameter sterilized pole-punch for each fungal strains, and transferred into 100 ml PDA sterilized liquid medium in a 250ml triangular flask. Fungi were shaken cultured at 28°C, 150 rpm, for 8 days. Mycelia of each fungal strain was harvested with sterilized cloth filter with several times wash using sterilized saline solution (0.9% NaCl). And the harvested mycelia were weighed and homogenized, and the homogenates were diluted into 1/100 (10 mg/mL) using sterilized saline solution. The prepared mycelium solutions were stored at 4°C for further use.

### Inoculation of fungal endophytes to grapevines

Approximately 160 physiologically similar vines in one vineyard were chosen and firstly divided into 3 blocks from south to north, and each block was then divided into 9 sub-blocks. Every sub-block containing 4 vines were inoculated with one fungal strain, forming one replicate of a treatment. Two rows in south and north ends, and two panel vines in west and east sides of the field were left untreated to avoid boundary effects. Eight strains of prepared fungal solutions and normal saline solution (as control) were painted to expanded vine leaves, 4 weeks after bud-burst (at least ten days before and after this inoculation, vines for inoculation were free of fungicide and pesticide). One vine at the end of every sub-block was left untreated as buffer between two different treatments within rows. The inoculations were done two times per day at morning and evening, respectively, for 3 days. In case it rained within 12 hours after each inoculation, an extra inoculation was conducted for compensation after the dry up of vine leaves. In total 60 mL fungal solution, approximately 600 mg mycelium was inoculated to each grapevine.

### Sampling

At berry maturity, berries and health full developed leaves (fungi inoculated and normal saline solution treated) were sampled for each sub-block. One or two clusters of berries and 5 randomly selected leaves for each vine in a sub-block were sampled. Berry and leaf samples were well packaged in ice boxes and delivered to lab within 4 hours for further analysis.

### Pre-treatment of leaf and berry samples

All leaf samples of each replicate were cut into approximately 1 cm^2^ pieces. Randomly selected ripen berries were picked off from clusters. Fresh samples were divided into 3 batches. One batch (more than 20 g for each sample) of leaf pieces and berries were homogenized into fresh fine powder in liquid nitrogen with a stainless grinder, stored at -80°C for reducing sugar, titratable acidity, total phenols, soluble protein and enzyme activity analysis. The second batch approximately 100 g samples were dried in wind-oven following a program of 110°C 10 min, 80°C 48 hours (72 hour for berries) and then were grounded into fine powder with a stainless grinder, for analyzing of total flavonoids, DPPH radical and superoxide anion scavenging capacities. The rest samples about 500 g fresh berry and 100 g of leaf were prepared and stored at -80°C for the extraction and determination of resveratrol.

### Determination of physio-chemical traits

For the determination of reducing sugar (RS), fresh fine powder (1 g) was added with 4 mL 1 mol/L zinc acetate (containing 3% glacial acetic acid) and 4 mL 0.25 mmol/L potassium ferrocyanide, and extracted at 80°C for 10 min with two times of vortex. The mixture was centrifuged at 5000 rpm and the supernatants was adjusted to pH = 7 by adding calcium carbonate powder. After 30 min of water bath at 60°C with several times of vortex, the solution was cooled to room temperature, and metered the volume to 10 mL with distilled water. After 10 min centrifuge at 5000 rpm, the supernatant was titrated with alkaline tartrate copper solution A+B [48]. The consumption of the supernatant was used to calculate the contents of RS. Total flavonoids (TF) content was determined using the aluminium chloride colorimetric method [49], with some modifications. Methanol extracts (0.5 mL), 10% aluminium chloride (0.1 mL), 1 mmol/L potassium acetate (0.1 mL) and distilled water (4.3 mL) were added after incubation at room temperature for 30 min. The absorbance was measured at 415 nm. TF content was calculated by comparing the results with rutin trihydrate as standard. Total phenols (TPh) were determined using the Forint phenol colorimetric method. Approximately 1 g fresh frozen powder was used for extraction and TPh were measured [50]. TPh content was standardized against gallic acid and expressed as milligrams per liter of gallic acid equivalents. For the determination of total soluble protein and antioxidant enzymes, fresh powder (1 g) was added to 10 mL of 0.1 mol/L potassium phosphate buffer (pH 7.0), containing 0.1 mmol/L EDTA-Na2, 0.5 mmol/L ascorbate and 1% PVPP (polyvinyl polypyrrolidone) and the mixture was incubated for 30 min with several rounds of vortex shaker. The mixture was centrifuged at 13,000 rpm at 4°C for 10 min. The supernatant was used to determine protein content and antioxidant enzyme activity. Total soluble protein (TPr) concentration was measured as described by Bradford [51] using bovine serum albumin as standard. Superoxide dismutase (SOD) was determined by the nitro-blue tetrazolium (NBT) method [52], and guaiacol peroxidase (GPX) assays were performed using the method described by Bergmeyer [53]. The extraction and determination of PAL was performed according to the method of Carolyn et al. [54], with some modifications as described by Xi et al. [55].

### Measurement of DPPH radical (DPPH) and Superoxide anion (SA) scavenging capacities

Dried sample was ground into fine powder, and weighed approximately 1 g into a volumetric flask. DPPH radical scavenging active substances were extracted by adding 50% of ethanol and sonicating for 30 min in an ultrasonic chamber. The mixture was filtered and the filtrate was diluted into gradient concentrations. DPPH radical scavenging capacity was measured and calculated according to Li et al. [56], absorbance was read at 517 nm in a spectrophotometer (S22, Biochrom Libra, England) and results were described as the percentages of DPPH radicals scavenged [56]. The prepared extracts gradient solutions for DPPH determination were also used for superoxide anion scavenging capacity (SA) measurement. And the determination of SA was following the method of Li et al. [55], and the SA scavenging capacity was described as the percentage of superoxide anions scavenged [55].

### Measurement for trans-resveratrol

Trans-resveratrol was extracted from homogenized 500 g fresh berries and 100 g fresh leaves with ethyl acetate. Two times extraction with 500 mL ethyl acetate was performed for both berry and leaf sample. Extracts were dried with rotary evaporator at 37°C and the dried extracts were dissolved in 1 mL ethanol. Trans-resveratrol was determined by HPLC method as described by Pascual-Marti et al. [57].

### Data analysis

Data were analysed using SPSS 16.0 software (SPSS Inc., Chicago, IL, USA) for Windows. One-way ANOVA followed by Tukey’s multiple comparison test at *P*<0.05, was used for the significance determination. Pearson’s correlation analysis was conducted to determine the correlations between variable. Response indexes (RI) were used to describe the effects of treatments and were calculated by the following formula: Response indexes (RI) = (V_treatment_-V_control_)/V_control_. In the formula, V_treatment_ is the mean value of a variable, and V_control_ is the mean value of control. A positive RI indicates promotion effect, and a negative RI indicates inhibition. Figures were plotted using Sigma Plot 10.0 (Systat Software Inc., San Jose, CA). Principal component analysis (PCA) and K-means clustering were performed using R software (R Development Core Team 2010). PCA was conducted on the mean-centered and scaled data in order to investigate the discriminations of the impacts of endophytic strains on leaves and berries of grapevines. K-means clustering was then employed to investigate the degree of similarity of physio-chemistry in responding to the inoculated fungal strains.

## Results

Compared to the controls, the detected physio-chemical traits could be significantly modified by one or more strains of the inoculated fungal endophytes, either in leaves or in berries (Table 2, S1 and S2 Tables). However, impacts of fungal inoculation were seen differently on leaves and on berries (Table 2). For some examples, in leaves, five of eight inoculated fungi (CXB-2, CXB-11, MXN-8, HCXL-16 and CXC-13) significantly promoted the content of reducing sugar (RS), but in berries, only two inoculated fungi (CXC-13 and CXC-9) appeared to promote this trait significantly. Additionally, inoculation of fungal strain CXB-2 tend to promote the leafy RS, but significantly decreased the berry RS (Table 2). Regarding TF, in leaves, seven inoculated fungal strains have significantly promoted, while in berries only four fungal strains had this effect (Table 2).

**Table 2:** Significance of difference between means of physio-chemical traits after treated with different strains of fungal endophytes.

Fungal strains	RS		TPr		TF		TPh		Res		PAL		GPX		SOD		DPPH		SA	
	L	B	L	B	L	B	L	B	L	B	L	B	L	B	L	B	L	B	L	B
**CXB-2**	**bc**	**c**	**a**	**ab**	**a**	**a**	**d**	**d**	**a**	**a**	**ab**	**ab**	**bc**	**bc**	**d**	**bc**	**ab**	**ab**	**a**	**ab**
**CXB-11**	**a**	**b**	**ab**	**ab**	**a**	**a**	**b**	**b**	**ab**	**b**	**ab**	**ab**	**abc**	**a**	**ab**	**c**	**c**	**a**	**a**	**a**
**MXN-8**	**cd**	**b**	**a**	**ab**	**b**	**a**	**ab**	**cd**	**a**	**bc**	**bc**	**b**	**c**	**c**	**cd**	**c**	**e**	**ab**	**ab**	**bcd**
**HCXL-16**	**bcd**	**b**	**a**	**ab**	**ab**	**c**	**b**	**a**	**b**	**de**	**a**	**a**	**c**	**bc**	**d**	**abc**	**d**	**ab**	**bc**	**cd**
**CXC-13**	**d**	**a**	**b**	**a**	**b**	**ab**	**ab**	**d**	**b**	**de**	**ab**	**ab**	**c**	**b**	**a**	**a**	**a**	**bc**	**cd**	**ef**
**Y73-11**	**e**	**b**	**b**	**b**	**a**	**abc**	**a**	**a**	**b**	**cde**	**d**	**ab**	**a**	**c**	**a**	**bc**	**a**	**ab**	**d**	**bc**
**HMC-7**	**de**	**b**	**c**	**b**	**c**	**bc**	**c**	**bc**	**b**	**bcd**	**bc**	**b**	**abc**	**a**	**bc**	**a**	**bc**	**d**	**d**	**f**
**CXC-9**	**e**	**a**	**c**	**b**	**ab**	**c**	**b**	**bc**	**b**	**de**	**bc**	**ab**	**ab**	**c**	**bc**	**ab**	**a**	**cd**	**bc**	**def**
**Control**	**e**	**b**	**c**	**b**	**c**	**c**	**c**	**cd**	**b**	**e**	**cd**	**b**	**abc**	**bc**	**ab**	**ab**	**d**	**bc**	**d**	**de**

One same letter existence means the two values are not different significantly. Otherwise, different letters means the values are significantly different (P<0.05). “L” represents the values of leaf and “B” represents the value of berry. Physio-chemical traits, RS: reducing sugar; TPr: soluble protein; TF: total flavonoid; TPh: total phenols; Res: content of trans-resveratrol; PAL: activity of phenylalanine ammonia-lyase; GPX: activity of Guaiacol peroxidase; SOD: activity of superoxide dismutase; DPPH: percentages of DPPH radical scavenged, at the concentration of 15ug/mL; SA: percentages of superoxide anion radical scavenged at the concentration of 10mg/mL.

**Fig 1:**
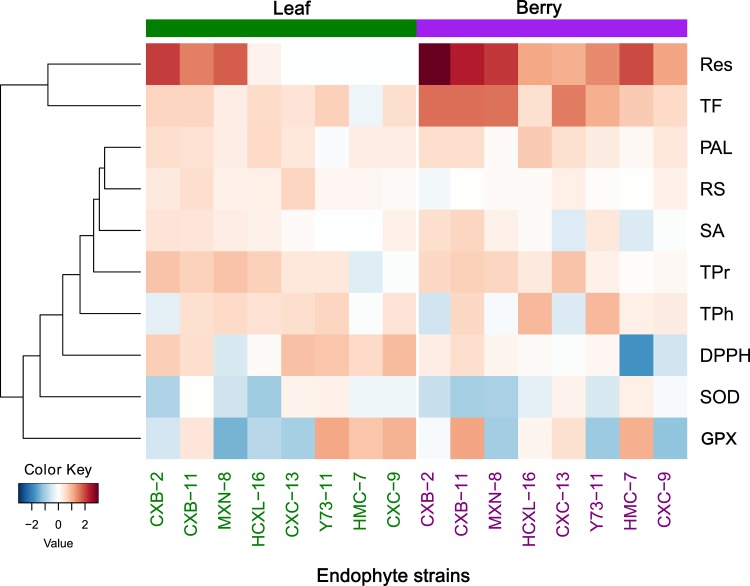
Heat-map and clustering of biochemical changes after the inoculation of different strains of fungal endophytes, in both leaves and berries of grapevines. Heat-map were drawn according to the response indexes (RI) values (S3 Table), and each row represents a biochemical variable and each column represents a fungal strain treatment. RI values were centered and scaled in the row direction to form virtual colors as presented in the color key. Biochemical parameters that showed a similar response to the inoculations of fungal endophytes were clustered together.

Response indexes (RI) were used to assess the impacts of certain inoculation of fungal endophytes on the physio-chemistries of grapevines. Compared to non-inoculation group (control), fungal inoculation have caused obviously changes to some detected biochemical traits, in both leaves and berries (Fig 1, Table 2 and S3 Table). Most of the physio-chemical traits (RS, TF, RES, PAL, DPPH and SA in vine leaves, as well as TPr, Res, and PAL in berries) were enhanced after the inoculation of fungal endophytes, as indicated as red colored or positive RI values (Fig 1, S3 Table). Physio-chemical traits TF, Res and PAL appeared positively responses to the endophytic inoculation, either in leaves and berries (Fig 1). Clustering analysis illustrated that all those detected physio-chemical traits could be divided into 3 groups: i) traits that have been greatly promoted by endophytic fungi, such as Res and TF; ii) traits that can be promoted or inhibited such as GPX, SOD, DPPH and others; and iii) traits that can hardly be influenced, such as RS, PAL, SA (Fig 1).

**Fig 2:**
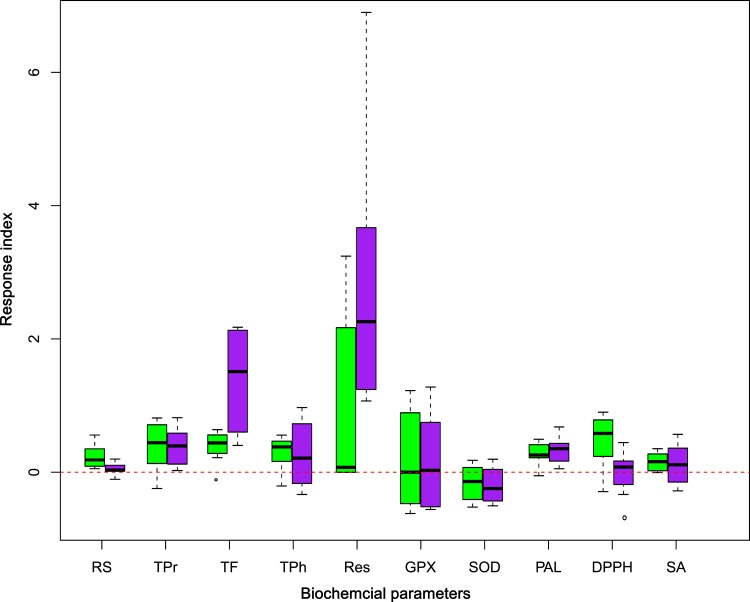
Boxplot of physio-chemical traits in response to fungal endophytes inoculations. Green colored boxes represent the leaf physio-chemical response ranges, and purple colored boxes represent the berry response ranges.

However, intensity of biochemical changes in grapevine varied among inoculations of different strains of fungal endophytes, and the RI variation boxplots displayed clearly the response ranges of the detected physio-chemical traits to fungal endophytes inoculations (Fig 2). Leafy TPr, Res, GPX, SOD, DPPH as well as berry TF, TPh, Res and GPX were modified in greater ranges by endophytes inoculation. Unlike these physio-chemical traits above, response ranges of RS, TF and PAL to the fungal inoculations were quite limited (Fig 2). Contents of secondary metabolites TF and Res can be greatly promoted by endophytes inoculation, both in leaves (~0.64 fold and 3.24 fold, respectively) and in berries (~ 2.18 fold and 6.9 fold, respectively) (S3 Table). On the other hand, activities of GPX and SOD in grapevines were promoted or inhibited by fungal inoculations. Similar effects were also observed on biochemical traits TPh, DPPH and SA after inoculated with different fungal strains (Fig 2, S3 Table).

**Fig 3:**
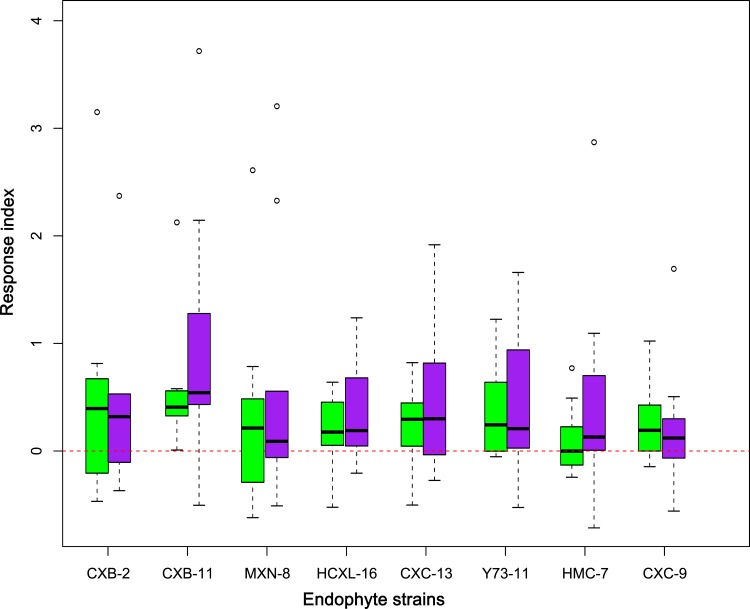
Boxplot of the impacts of inoculated fungal strains on grapevine physio-chemical traits. Green colored boxes represent the impacts on leaf biochemistries, and purple colored boxes represent the impacts on berry biochemistries.

Additionally, RI variation boxes showed the inoculation of endophytic fungal strains CXB-2, CXB-11 and CXC-13 conferring great potentials in modifying the detected physio-chemical traits (Fig 3). In leaves, inoculation of endophytic fungal strains CXB-11 and CXB-2 changed 9 and 8, respectively, of the total 10 detected physio-chemical traits in greater ranges (RI>0.3) (Fig 3, S3 Table). And in berries, the inoculation of these two fungal strains had also changed 8 and 7 respectively of the berry physio-chemical traits in greater ranges (RI>0.3) (Fig 3, S3 Table). Inoculation of other endophytic fungi strains had only modified 4–6 of the detected biochemical traits in greater ranges (RI>0.3) (S3 Table). In leaves, Inoculation of fungal strains CXB-2, MXN-8 and Y73-11 had triggered greater ranges of physio-chemical vibrations, and in berries, fungal strains CXB-11, CXC-13 and Y73-11 induced greater ranges of metabolism variations (Fig 3). Conversely, inoculation of fungal strain CXC-9 caused lesser impacts on grapevines’ physio-chemistries, either in leaves or in berries (Fig 3).

**Fig 4:**
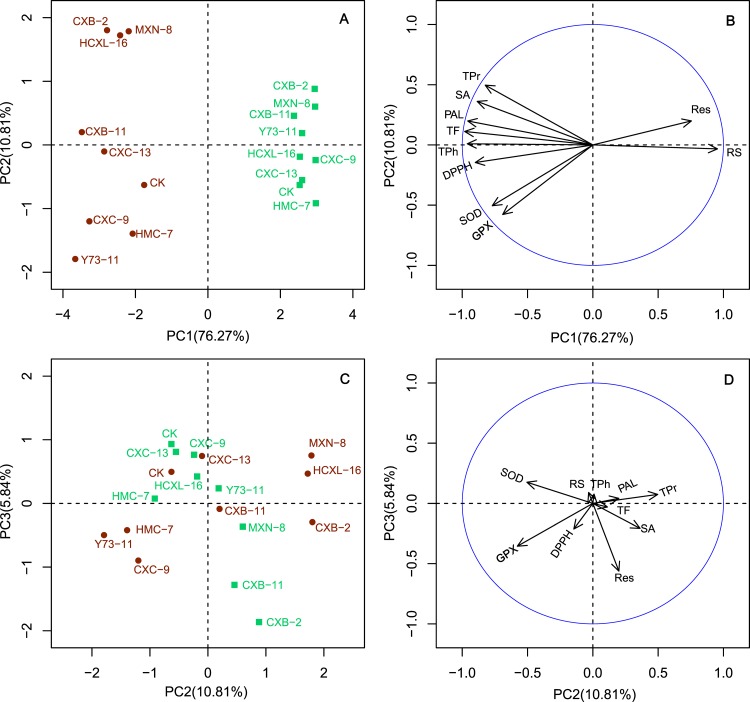
Principal component analysis (PCA) of the impacts of endophytic fungal inoculation on leaves and berries of grapevines. A and C are discriminations of leaves (green) and berries (red) after inoculated with different fungal strains. B is loading plot of biochemical parameters for the first two principle components, PC1 and PC2. D is loading plot of biochemical parameters for the second and third principle components, PC2 and PC3.

PCA (principal component analysis) of all these data provided an overview for the effects of endophytic fungi inoculation on grape biochemical properties of both the berry and leaf samples (Fig 4). The first three principal components (PC1, PC2 and PC3) explained about 93% of the total variances. PC1 mainly separated berries and leaves, based largely the contents of reducing sugar (RS), total phenols (TPh), total flavonoids (TF) and phenylalanine-lyase activity (PAL). Physio-chemical parameters GPX, SOD, PAL, DPPH, SA, TF and TPh were all higher in leaves than in berries (S1 and S2 Tables). Leaf samples contain almost 10 times of TF and TPh contents than that of in berries. Conversely, grape berries contain 100 times of trans-resveratrol (Res) and 2–3 times of reducing sugars (RS) than that of leaves (S1 and S2 Tables). PC2 resolved the effects of different fungal strains into 3 clusters in berries, related mainly to the differences of total soluble protein (TPr), superoxide scavenging capacity (SA), guaiacol peroxidase (GPX), and superoxide dismutase (SOD) activities. The first cluster included fungal strains of CXB-2, HCXL-16 and MXN-8, which related mainly to the influences of berry TPr and SA; the second cluster included CXB-11, CXC-13 and control; and the other cluster included the rest inoculated fungal trains CXC-9, HMC-7 and Y73-11, mainly related to the modified activities of SOD and GPX (Fig 4A and 4B). Principal components PC3 further separated the effects on leaves into two groups mainly by the different of resveratrol content (Res) (Fig 4C & 4D).

In current experiment, endophytic fungi were inoculated to vine leaves, however, physio-chemical changes were detected in both leaves and berries at maturity stage. Quantitative correlations of those detected parameters were tested within (and between) leaves and berries, and coefficients of correlations were shown in Table 3 and Table 4, respectively. Only 5 in leaves and 4 in berries, respectively, of the inter-parameter pairs were tested significant correlation (Table 3). In contrast, 18 parameter pairs were tested significant correlations (P<0.05) between leaves and berries. Amongst, TPr, Res, PAL and SA were changed coordinately in significant correlation between leaf and berry (Table 4). Other correlations deserve to mention were leafy RS significantly correlated with berry TPr and TF; leafy TPr significantly correlated with berry TPr, TF, DPPH and SA simultaneously, and negatively correlated with berry SOD; leafy TF significantly correlated with berry PAL, DPPH and SA; and leafy Res significantly correlated with berry TF, Res, SA simultaneously; and the changes of leafy SA also significantly correlated to berry Res. On the other hand, berry SA significantly correlated with leafy TPr, TF, Res and SA; and berry SOD significantly but negatively correlated with leafy TPr, RES, and SA simultaneously (Table 4). Correlation analysis provides clues in interpreting the co-vibrations of some physio-chemical traits between leaves and berries in this experiment.

**Table 3:** Coefficients of correlations among detected parameters within leaves (left-below) or berries (right-above) of grapevines.

	RS	TPr	TF	TPh	Res	GPX	SOD	PAL	DPPH	SA
**RS**		**0.185**	**-0.097**	**-0.004**	**-0.636**	**-0.12**	**0.422**	**0.083**	**-0.255**	**-0.561**
**TPr**	**0.452**		0.880**	**-0.375**	**0.417**	**0.191**	**-0.36**	**0.393**	**0.583**	**0.278**
**TF**	**0.371**	**0.602**		**-0.384**	0.710*	**0.154**	**-0.548**	**0.181**	**0.541**	**0.493**
**TPh**	**0.227**	**0.18**	**0.416**		**-0.319**	**-0.004**	**-0.212**	**0.365**	**0.104**	**0.218**
**Res**	**0.288**	0.769*	**0.299**	**-0.253**		**0.183**	**-0.55**	**0.18**	**0.315**	**0.606**
**GPX**	**-0.481**	**-0.628**	**0.071**	**0.097**	**-0.453**		**0.198**	**0.138**	**-0.078**	**-0.04**
**SOD**	**0.134**	**-0.514**	**-0.025**	**0.348**	**-0.507**	**0.498**		**-0.211**	-0.792*	-0.909**
**PAL**	**0.551**	**0.519**	**0.276**	**-0.159**	**0.427**	**-0.507**	**-0.654**		**0.382**	**0.26**
**DPPH**	**0.249**	**-0.326**	**0.394**	**-0.009**	**-0.242**	**0.542**	**0.427**	**-0.008**		0.811**
**SA**	**0.385**	0.744*	**0.513**	**-0.117**	0.853**	**-0.364**	**-0.56**	0.702*	**-0.085**	

** correlation is significant at the 0.01 level

* Correlation is significant at the 0.05 level (2-tailed).

**Table 4:** Coefficients of correlations of the detected parameters between leaf (xx.l) and berry (xx.b) of grapevines.

	RS.b	TPr.b	TF.b	TPh.b	Res.b	POD.b	SOD.b	PAL.b	DPPH.b	SA.b
**RS.l**	**0.27**	0.922**	0.733*	**-0.267**	**0.295**	**0.449**	**-0.116**	**0.524**	**0.444**	**0.136**
**TPr.l**	**-0.322**	0.672*	0.675*	**-0.006**	**0.571**	**-0.17**	-0.811**	**0.482**	0.805**	0.673*
**TF.l**	**-0.004**	**0.413**	**0.424**	**0.359**	**0.307**	**-0.27**	**-0.608**	0.692*	0.674*	0.678*
**TPh.l**	**0.648**	**0.279**	**0.153**	**0.488**	**-0.421**	**-0.247**	**-0.254**	**0.186**	**0.273**	**0.053**
**Res.l**	**-0.559**	**0.538**	0.759*	**-0.404**	0.881**	**-0.026**	-0.749*	**0.072**	**0.561**	0.688*
**POD.l**	**0.057**	**-0.619**	**-0.379**	**0.448**	**-0.185**	**-0.025**	**0.182**	**-0.226**	**-0.404**	**0.006**
**SOD.l**	**0.41**	**-0.017**	**-0.038**	**0.063**	**-0.45**	**0.169**	**0.33**	**-0.328**	**-0.052**	**-0.152**
**PAL.l**	**-0.085**	**0.495**	**0.366**	**-0.068**	**0.498**	**0.384**	**-0.225**	0.748*	**0.213**	**0.178**
**DPPH.l**	**0.29**	**0.055**	**0.097**	**-0.048**	**0.089**	**0.043**	**0.332**	**0.282**	**-0.227**	**-0.084**
**SA.l**	**-0.348**	**0.529**	**0.634**	**-0.173**	0.771*	**0.078**	-0.749*	**0.442**	**0.596**	0.721*

**: correlation is significant at the 0.01 level

*: Correlation is significant at the 0.05 level (2-tailed).

## Discussion

Currently, fewer studies were covered the influences of endophytic fungi on grape metabolites. However, interactions existed between endophytes and plant pathogens, as well as endophytes and their host plants have some reports [58,59]. But the underlying mechanisms are still not well understood. As raw materials of some organoleptic sensitive products such as wine grape, since subtle changes of metabolites in grape berries will cause significant sensory effects to the resultant products [60]. Therefore, the biochemical impacts of endophytes on wine grape should be considered. This study initially designed to investigate the biochemical impacts of exogenous endophytic fungi on grapevines. And all vines chosen for treatment are within one vineyard with same cultivar and age, with similar physiological status, as well as containing similar fungal endophytes communities [47]. The inoculation of exogenous fungal endophytes had led to four impacts on the endophytic fungal communities of the treated vines: i) inoculated fungus successfully infected and became the dominant endophytes within the infected grapevines; ii) inoculated endophytic strains have infected vines but not become dominant endophytic fungal species in the treated vines; and iii) re-inoculated fungus not detected from the inoculated plant ([47] and see summarized in Table 1) In any cases, all these results confer effects in reconstructing the endophytic communities of the treated vines [47]. Therefore, the detected changes of vine metabolites in this experiment were actually the consequences of both the inoculations and the altered endogenous endophytic communities.

Whatever happened during the process, the inoculation of different strains of fungal endophytes in this experiment has reshaped grapevines’ physio-chemistry and created different metabolic status of the target grapevines (Fig 1). In particular, endophytic strains CXB-11, CXC-13 and Y73-11 detected stronger promotion effects on grape metabolic regulation than other fungal strains, either in leaves or in berries (Fig 3). Fungal strains CXB-11 and Y73-11 belong to genus *Nigrospora* and *Colletotrichum sp*, respectively, are dominant species within the host vines leaves, and had successfully infected and become dominant endophytic fungus within the treated vines (Table 1). Interestingly enough, CXC-13 was a rare isolated endophytic fungal strains, but its inoculation had also initiated greater effects on grapevines (Fig 3). However, relationships between fungal features and its metabolic re-shaping abilities need further studies. But the fact that inoculation of different strains of fungal endophytes led to different grape metabolites status, implies the possibilities to apply different endophytes or endophytic combinations to create different characters, styles and qualities of wine grape and the resultant wine. One may even purposely manage vineyards by continuous training with certain kind of endophyte or endophytic combinations to create vines containing designed endophytic community structures for sustainable production of quality and characteristic wine grapes.

The French term “terroir” which endued a certain wine of distinguished characters, includes the local soil, climate factors, ambient organisms and all other [61]. We currently argue that the roles of endophytes in “terroir” should be taken into account. Additionally, whether endophytes have partially mediated the impacts between climate factors and grapevines needs to be re-evaluated, since endophytes communities within certain plant are influenced by climate and other geographic factors [62,63]. Due to the limited biomass of endophytes in natural growing plants [64], their metabolic impacts on host plant should also be limited, as comparison to genotype and other environmental factors such as soil, nutrients, radiation, temperature, precipitation. [65–67]. However, subtle change of metabolites, especially secondary metabolites of wine grape, was enough to influence the organoleptic style of the resultant wine. These secondary metabolites such as phenols, flavonoids, tannins, terpenes, stilbenes and others, confer multiple sensory characters to wine and exert health protective effects to human being [68–71]. Therefore, delicate use of endophytic fungi may become a good fine-tuning tool for regulating wine grape secondary metabolites. But how to use this tool to reach our targeted metabolite patterns of grape berries warrants further researches.

While physio-chemical modifications were detected in both leaf and berry at berry maturity stage. The mechanism which lies in this phenomenon may be the continuous exchange of metabolites and signals between leaves and berries. It is known that exchanges of transportable metabolites, signal molecules, as well as some defense compounds among different parts of plant are occurring continuously [72]. Biochemical traits that significant correlation in quantitative between grape berries and leaves were partially explained the coordinated responses of those biochemical traits to endophytic inoculations in both leaves and berries (Table 4). Coordinate responses of some metabolites simultaneous in both the fruits and leaves of grapevine have had evidences [73], and furtherly implies the possibility to shape berry metabolites by means of leaf treatments.

While discussing the prospects of this fine-turning regulator to shape wine grape metabolites, negative effects should also be considered, such as risks of producing harmful or toxic compounds. However, this could be avoided by using endophytic strains isolated from local vineyards, and following a systematic research for each candidate fungal endophytes before it can be used in viticulture. Endophytes can occasionally become pathogens [74], but compared to pathogens or other source of fungi, endophytes are still a safe tool for use.

## Conclusion

Inoculation of fungal endophytes to leaves in earlier stages can alter grapevine physio-chemical status in both leaves and berries at berry ripening stage. Some endophytic fungal strains such as CXB-11, CXC-13 and Y73-11 greater ranges of impacts on grapevine metabolites. Inoculation of different strains of fungal endophytes created different patterns of biochemical status of grape. Results imply the possibilities using fungal endophytes as fine-tuning regulators to shape the qualities and characters of wine grape.

## Supporting Information

S1 Table Results of detected physio-chemical traits in leaves of grapevine after treated by different strains of fungal endophytes. Values in the table are illustrated as means ± standard errors. At least one same letter existence means the values are not different significantly. Otherwise, different letters means the values are significantly different (P<0.05). Physio-chemical traits, RS: reducing sugar; TPr: soluble protein; TF: total flavonoid; TPh: total phenols; Res: content of trans-resveratrol; PAL: activity of phenylalanine ammonia-lyase; GPX: activity of Guaiacol peroxidase; SOD: activity of superoxide dismutase; DPPH: percentages of DPPH radical scavenged, at the concentration of 15ug/mL; SA: percentages of superoxide anion radical scavenged at the concentration of 10mg/mL.
(PDF)

S2 Table Results of detected physio-chemical traits in berries of grapevine after treated by different strains of fungal endophytes. Values in the table are illustrated as means ± standard errors. One same letter existence means the values are not different significantly. Otherwise, different letters means the values are significantly different (P<0.05). Physio-chemical traits, RS: reducing sugar; TPr: soluble protein; TF: total flavonoid; TPh: total phenols; Res: content of trans-resveratrol; PAL: activity of phenylalanine ammonia-lyase; GPX: activity of Guaiacol peroxidase; SOD: activity of superoxide dismutase; DPPH: percentages of DPPH radical scavenged, at the concentration of 15ug/mL; SA: percentages of superoxide anion radical scavenged at the concentration of 10mg/mL.
(PDF)

S3 Table Response indexes (RI) of physio-chemical traits in grapevine leaves caused by different strains of fungal endophytes. Positive value represents the treatment initiated a promotion effect to the corresponding biochemical trait, and negative value means the fungal strain caused an inhibition effect to the corresponding trait of grape vine.
(PDF)

S4 Table Response indexes (RI) of physio-chemical traits in grapevine berries caused by different strains of fungal endophytes. Positive value represents the treatment initiated a promotion effect to the corresponding biochemical trait, and negative value means the fungal strain caused an inhibition effect to the corresponding trait of grape vine.
(PDF)
