# Supplementary material for: Fungal Endophytes as a Metabolic Fine-Tuning Regulator for Wine Grape
Source: PLoS One. 2016 Sep 22;11(9):e0163186. doi: 10.1371/journal.pone.0163186 (PMC5033586; doi:10.1371/journal.pone.0163186)
Supplement: S1 Table — Values in the table are illustrated as means ± standard errors. At least one same letter existence means the values are not different significantly. Otherwise, different letters means the values are significantly different (P<0.05). Physio-chemical traits, RS: reducing sugar; TPr: soluble protein; TF: total flavonoid; TPh: total phenols; Res: content of trans-resveratrol; PAL: activity of phenylalanine ammonia-lyase; GPX: activity of Guaiacol peroxidase; SOD: activity of superoxide dismutase; DPPH: percentages of DPPH radical scavenged, at the concentration of 15ug/mL; SA: percentages of superoxide anion radical scavenged at the concentration of 10mg/mL. (PDF) [file pone.0163186.s001.pdf]

S1 Table. Results of detected physio-chemical traits in leaves of grapevine after treated by different strains of fungal endophytes

fungal strain	mg/gFW RS	mg/gFW TPr	mg/gDW TF	mg/gFw TPh	μg/gFW Res	U/(gFW .min) PAL	U/(gFW.min) GPX	U/(gFW .min)) SOD	% DPPH	% SA
**CXB-2**	**54.69±0.87bc**	**11.68±0.22a**	**26.37±1.31a**	**2.08±0.12d**	**6.66±1.73a**	**219.22±21.33ab**	**93.33±15.72bc**	**271.32±82.72d**	**69.81±4.68ab**	**70.2±1.53a**
**CXB-11**	**60.91±3.09a**	**10.17±0.24ab**	**26.33±0.39a**	**3.65±0.06b**	**4.45±1.92ab**	**212.44±14.94ab**	**180±20abc**	**514.73±11.71ab**	**59.35±2.53c**	**68.83±0.76a**
**MXN-8**	**50.27±1.94cd**	**11.5±0.91a**	**20.57±0.38b**	**3.89±0.34ab**	**5.49±0.94a**	**184.56±21.45bc**	**52±3.46c**	**331.78±60.52cd**	**29.62±1.16e**	**63.38±1.26ab**
**HCXL-16**	**51.03±0.33bcd**	**10.56±0.98a**	**24.55±0.44ab**	**3.58±0.13b**	**1.8±0.78b**	**228.22±5.87a**	**76.67±25.17c**	**243.41±32.67d**	**43.95±5.34d**	**60.11±2.16bc**
**CXC-13**	**66.64±3.28d**	**8.37±0.74b**	**22.73±0.68b**	**3.79±0.06ab**	**1.57±0.9b**	**197±14.38ab**	**68±8c**	**578.29±18.8a**	**76.04±1.85a**	**54.22±5.58cd**
**Y73-11**	**46.58±1.24de**	**8.42±0.43b**	**27.68±0.82a**	**4.08±0.01a**	**1.57±0.36b**	**144.56±2.22d**	**304±141.32a**	**601.55±11.7a**	**73.06±1.93a**	**51.6±2.08d**
**HMC-7**	**46.62±1.09de**	**4.87±0.41c**	**14.41±0.88c**	**2.53±0.14c**	**1.57±0.27b**	**187.11±4.53bc**	**240.67±133.17abc**	**443.41±33.65bc**	**62.29±3.89bc**	**51.82±3.11d**
**CXC-9**	**45.01±1.03e**	**6.19±0.78c**	**24.1±1.83ab**	**3.56±0.11b**	**1.57±0.63b**	**187.33±5.04bc**	**276.33±21.01ab**	**435.66±28.04bc**	**79.34±5.15a**	**60.07±2.37bc**
**Control**	**42.76±0.88e**	**6.44±0.57c**	**16.89±1.3c**	**2.62±0.04c**	**1.57±0.21b**	**152.67±10.04cd**	**136.67±4.62abc**	**510.08±14.21ab**	**41.75±0.62d**	**51.88±2.46d**

Values in the table are illustrated as means ± standard errors. At least one same letter existence means the values are not different significantly. Otherwise, different letters means the values are significantly different ($P<0.05$). Physio-chemical traits, RS: reducing sugar; TPr: soluble protein; TF: total flavonoid; TPh: total phenols; Res: content of trans-resveratrol; PAL: activity of phenylalanine ammonia-lyase; GPX: activity of Guaiacol peroxidase; SOD: activity of superoxide dismutase; DPPH: percentages of DPPH radical scavenged, at the concentration of 15ug/mL; SA: percentages of superoxide anion radical scavenged at the concentration of 10mg/mL.
