# Supplementary material for: Fungal Endophytes as a Metabolic Fine-Tuning Regulator for Wine Grape
Source: PLoS One. 2016 Sep 22;11(9):e0163186. doi: 10.1371/journal.pone.0163186 (PMC5033586; doi:10.1371/journal.pone.0163186)
Supplement: S2 Table — Values in the table are illustrated as means ± standard errors. One same letter existence means the values are not different significantly. Otherwise, different letters means the values are significantly different (P<0.05). Physio-chemical traits, RS: reducing sugar; TPr: soluble protein; TF: total flavonoid; TPh: total phenols; Res: content of trans-resveratrol; PAL: activity of phenylalanine ammonia-lyase; GPX: activity of Guaiacol peroxidase; SOD: activity of superoxide dismutase; DPPH: percentages of DPPH radical scavenged, at the concentration of 15ug/mL; SA: percentages of superoxide anion radical scavenged at the concentration of 10mg/mL. (PDF) [file pone.0163186.s002.pdf]

S2 Table. Results of detected physio-chemical traits in berries of grapevine after treated by different strains of fungal endophytes

fungal strain	mg/gFW RS	mg/gFW TPr	mg/gDW TF	mg/gFw TPh	μg/gFW Res	U/(gFW .min) PAL	U/(gFW.min) GPX	U/(gFW .min)) SOD	% DPPH	% SA
**CXB-2**	**115.14±0.322c**	**4.27±0.32ab**	**3.08±0.38a**	**0.22±0.02d**	**78.21±1.77a**	**41.72±2.7ab**	**40±20.25bc**	**165.89±51.86bc**	**23.34±1.63ab**	**41.13±1.54ab**
**CXB-11**	**131.26±0.72b**	**4.51±0.91ab**	**3.05±0.4a**	**0.5±0.06b**	**49.7±9.82b**	**41.67±0.93ab**	**97.47±4.4a**	**130.23±66.23c**	**27.92±6.05a**	**45.11±0.59a**
**MXN-8**	**135.09±2.17b**	**4.34±0.74ab**	**3.02±0.52a**	**0.31±0.01cd**	**42.74±7.36bc**	**30.61±1.29b**	**20.97±1.89c**	**133.33±12.81c**	**21.79±2.3ab**	**33.83±1.26bcd**
**HCXL-16**	**134.20±1.67b**	**3.51±0.68ab**	**1.36±0.57c**	**0.65±0.08a**	**22.16±3.25de**	**48.89±2.55a**	**481±8.52bc**	**208.53±11.47abc**	**20.45±2.48ab**	**30.1±2.16cd**
**CXC-13**	**154.14±1.62a**	**5.07±0.24a**	**2.83±0.61ab**	**0.24±0.04d**	**20.48±3.62de**	**40.89±2.18ab**	**60.03±3.01b**	**302.17±13.67a**	**18.65±0.88bc**	**21.14±5.58ef**
**Y73-11**	**132.18±0.73b**	**3.24±0.69b**	**2.04±0.59abc**	**0.64±0.02a**	**26.33±6.32cde**	**36.5±2.85ab**	**20.32±3.39c**	**188.56±50.56bc**	**21.17±0.31ab**	**37.11±2.08bc**
**HMC-7**	**129.69±0.78b**	**2.86±1.13b**	**1.65±0.37bc**	**0.39±0bc**	**38.2±3.27bcd**	**31.39±13.14b**	**89.57±1.3a**	**314.01±17.76a**	**5.51±2.72d**	**20.72±3.11f**
**CXC-9**	**149.20±0.90a**	**3.02±0.43b**	**1.46±0.21c**	**0.41±0.02bc**	**22.21±2.15de**	**37.83±1.09ab**	**18.88±11.34c**	**245.36±20.68ab**	**12.88±0.64cd**	**27.92±2.37def**
**Control**	**128.73±0.32b**	**2.79±0.57b**	**0.97±0.24c**	**0.33±0.05cd**	**9.9±4.73e**	**29.1±3.55b**	**42.78±5.1bc**	**262.72±45.16ab**	**19.32±1.7bc**	**28.75±2.47de**

Values in the table are illustrated as means ± standard errors. One same letter existence means the values are not different significantly. Otherwise, different letters means the values are significantly different (P<0.05). Physio-chemical traits, RS: reducing sugar; TPr: soluble protein; TF: total flavonoid; TPh: total phenols; Res: content of trans-resveratrol; PAL: activity of phenylalanine ammonia-lyase; GPX: activity of Guaiacol peroxidase; SOD: activity of superoxide dismutase; DPPH: percentages of DPPH radical scavenged, at the concentration of 15ug/mL; SA: percentages of superoxide anion radical scavenged at the concentration of 10mg/mL.
