# Supplementary material for: Fungal Endophytes as a Metabolic Fine-Tuning Regulator for Wine Grape
Source: PLoS One. 2016 Sep 22;11(9):e0163186. doi: 10.1371/journal.pone.0163186 (PMC5033586; doi:10.1371/journal.pone.0163186)
Supplement: S3 Table — Positive value represents the treatment initiated a promotion effect to the corresponding biochemical trait, and negative value means the fungal strain caused an inhibition effect to the corresponding trait of grape vine. (PDF) [file pone.0163186.s003.pdf]

S3 Table. Response indexes (RI) of physio-chemical traits of grapevine leaves caused by different strains of fungal endophytes

Fungal strain	RS	TPr	TF	TPh	Res	GPX	SOD	PAL	DPPH	SA
CXB-2	0.28	**0.81**	**0.56**	-0.21	**3.24**	**-0.32**	**-0.47**	**0.44**	**0.67**	**0.35**
CXB-11	**0.42**	**0.58**	**0.56**	**0.39**	**1.84**	**0.32**	0.01	**0.39**	**0.42**	**0.33**
MXN-8	0.18	**0.79**	0.22	**0.48**	**2.50**	**-0.62**	**-0.35**	0.21	-0.29	0.22
HCXL-16	0.19	**0.64**	**0.45**	**0.37**	0.15	**-0.44**	**-0.52**	**0.49**	0.05	0.16
CXC-13	**0.56**	**0.30**	**0.35**	**0.45**	0.00	**-0.50**	0.13	0.29	**0.82**	0.05
Y73-11	0.09	**0.31**	**0.64**	**0.56**	0.00	**1.22**	0.18	-0.05	**0.75**	-0.01
HMC-7	0.09	-0.24	-0.15	-0.03	0.00	**0.76**	-0.13	0.23	**0.49**	0.00
CXC-9	0.05	-0.04	**0.43**	**0.36**	0.00	**1.02**	-0.15	0.23	**0.90**	0.16

Positive value represents the treatment initiated a promotion effect to the corresponding biochemical trait, and negative value means the fungal strain caused an inhibition effect to the corresponding trait of grape vine.
