# Supplementary material for: Fungal Endophytes as a Metabolic Fine-Tuning Regulator for Wine Grape
Source: PLoS One. 2016 Sep 22;11(9):e0163186. doi: 10.1371/journal.pone.0163186 (PMC5033586; doi:10.1371/journal.pone.0163186)
Supplement: S4 Table — Positive value represents the treatment initiated a promotion effect to the corresponding biochemical trait, and negative value means the fungal strain caused an inhibition effect to the corresponding trait of grape vine. (PDF) [file pone.0163186.s004.pdf]

S4 Table. Response indexes (RI) of physio-chemical traits of grapevine berries caused by different strains of fungal endophytes

Fungal strain	RS	TPr	TF	TPh	Res	GPX	SOD	PAL	DPPH	SA
CXB-2	-0.11	**0.53**	**2.18**	**-0.33**	**6.90**	-0.06	**-0.37**	**0.43**	0.21	**0.43**
CXB-11	0.02	**0.62**	**2.14**	**0.52**	**4.02**	**1.28**	**-0.50**	**0.43**	**0.45**	**0.57**
MXN-8	0.05	**0.56**	**2.11**	-0.06	**3.32**	**-0.51**	**-0.49**	0.05	0.13	0.18
HCXL-16	0.04	0.26	**0.40**	**0.97**	**1.24**	0.12	-0.21	**0.68**	0.06	0.05
CXC-13	**0.20**	**0.82**	**1.92**	-0.27	**1.07**	**0.40**	0.15	**0.41**	-0.03	-0.26
Y73-11	0.03	0.16	**1.10**	**0.94**	**1.66**	**-0.53**	-0.28	0.25	0.10	0.29
HMC-7	0.01	0.03	**0.70**	0.18	**2.86**	**1.09**	0.20	0.08	**-0.71**	-0.28
CXC-9	0.16	0.08	**0.51**	0.24	**1.24**	**-0.56**	-0.07	**0.30**	**-0.33**	-0.03

Positive value represents the treatment initiated a promotion effect to the corresponding biochemical trait, and negative value means the fungal strain caused an inhibition effect to the corresponding trait of grape vine.
